# Recent advances in biomarkers for diabetes mellitus and tuberculosis comorbidity: a comprehensive review

**DOI:** 10.3389/fendo.2025.1630603

**Published:** 2025-08-14

**Authors:** Lihua Fang, Yuqian Wu, Xiaokang Fang, Jie Ning

**Affiliations:** ^1^ Department of Endocrinology, Shenzhen Longhua District Central Hospital, Shenzhen, Guangdong, China; ^2^ Guangzhou Center for Disease Control and Prevention, Guangzhou, Guangdong, China

**Keywords:** diabetes mellitus, tuberculosis, comorbidity, biomarkers, advancements

## Abstract

Diabetes mellitus (DM) and tuberculosis (TB) are significant global health challenges that complicate diagnosis, treatment, and management due to their interrelated nature. DM increases TB risk and worsens outcomes, highlighting the need for early detection and effective management. This review summarizes recent advancements in biomarkers for DM-TB comorbidity, including microbial, metabolic, immunological, inflammatory, clinical, and genetic markers. We identified 30 relevant studies, through a literature search using keywords related to DM, TB, and biomarkers. Key findings include specific gut microbiota genera and lipid mediators that show promise for early diagnosis and treatment. Immunological biomarkers like altered CD8+ T cells and NK cells provide insights into disease severity and treatment monitoring. Inflammatory markers such as elevated CRP, ferritin, and IL-6 reflect heightened inflammation and could guide treatment strategies. Clinical biomarkers, including serum CA-125 (sensitivity 88.14%, specificity 95.83%) and AUC/MIC ratios of anti-TB drugs (e.g., moxifloxacin ≥67; sensitivity 97.3%, specificity 90.0%), demonstrate high diagnostic accuracy. Future research should focus on validating these biomarkers across diverse populations and integrating them into clinical practice to enhance DM-TB management and contribute to global disease control efforts.

## 1 Introduction

Diabetes mellitus (DM) and Tuberculosis (TB) are two major global health challenges that significantly impact each other, posing a complex and growing public health concern (1). The global prevalence of diabetes mellitus, which affected around 425 million people in 2017, is projected to soar to 629 million individuals by 2045, posing significant medical and social challenges (2). DM including both insulin-dependent diabetes mellitus (IDDM) and non-insulin-dependent diabetes mellitus (NIDDM), significantly escalates the propensity for contracting TB by nearly threefold, while also augmenting the risk of mortality during TB treatment and exacerbating the incidence of adverse therapeutic outcomes (3, 4). In a population of nearly 90,000 individuals with diabetes, they identified a tuberculosis prevalence rate of 179 cases per 100,000 persons in Jiangshu, China (5, 6). The increased risk of TB in DM patients is attributed to the immuno-suppressive effects of hyperglycemia, which impairs the body’s ability to fight off infections. Moreover, DM is associated with more severe disease presentation, delayed sputum conversion, and higher rates of treatment failure in TB patients (7). The presence of DM complicates the management of TB, particularly in cases of multidrug-resistant TB (MDR-TB) (8), where adherence to treatment regimens is crucial. Studies have shown that DM is linked to higher rates of MDR-TB, further complicating treatment and management (9). Moreover, DM patients with TB often experience prolonged treatment durations, lower sputum conversion rates, and higher mortality rates (10). This emphasizes the need for improved diagnostic tools and treatment strategies to address the unique challenges posed by the comorbidity of these diseases.

For diabetes, various biomarkers have been identified to aid in diagnosis and management. These include metabolic markers such as glycated hemoglobin (HbA1c) and fasting blood glucose levels, which are critical for monitoring glycemic control (11). Additionally, immune markers such as cytokines and chemokines have been studied for their potential roles in diabetes pathogenesis and treatment response (12). In contrast, the diagnosis of tuberculosis remains challenging due to the limitations of traditional methods such as culture and smear microscopy. These methods are slow and often lack sensitivity, particularly in settings with high disease burden. Molecular techniques like GeneXpert MTB/RIF have improved diagnostic capabilities but are costly and require significant infrastructure. As highlighted by MacLean et al. (2019) (13), there is a need for more accessible and affordable biomarkers for TB diagnosis. They identified several promising biomarkers, including host-based markers like cytokines, antibodies, and RNA signatures, as well as pathogen-based markers such as lipoarabinomannan (LAM). However, the validation and implementation of these biomarkers have been slow, and few have reached the necessary diagnostic performance criteria set by the World Health Organization (WHO).

In the context of comorbid DM and TB, the identification of shared biomarkers is crucial. These biomarkers could serve as diagnostic tools, aid in monitoring disease progression, and guide treatment decisions. Despite significant progress in understanding the pathophysiological mechanisms underlying the comorbidity of DM and TB (14), there remains a gap in validated biomarkers for both diseases, especially in resource-limited settings. By synthesizing recent findings from the past decade here, we stress the potential clinical applications of these markers in improving the diagnosis, monitoring, and treatment of DM-TB comorbidity. These markers span across microbial and metabolic, immunological, inflammatory response, clinical and diagnostic, genetic and molecular domains, offering a multifaceted approach to understanding and managing this complex comorbidity.

## 2 Materials and methods

**Figure 1:**
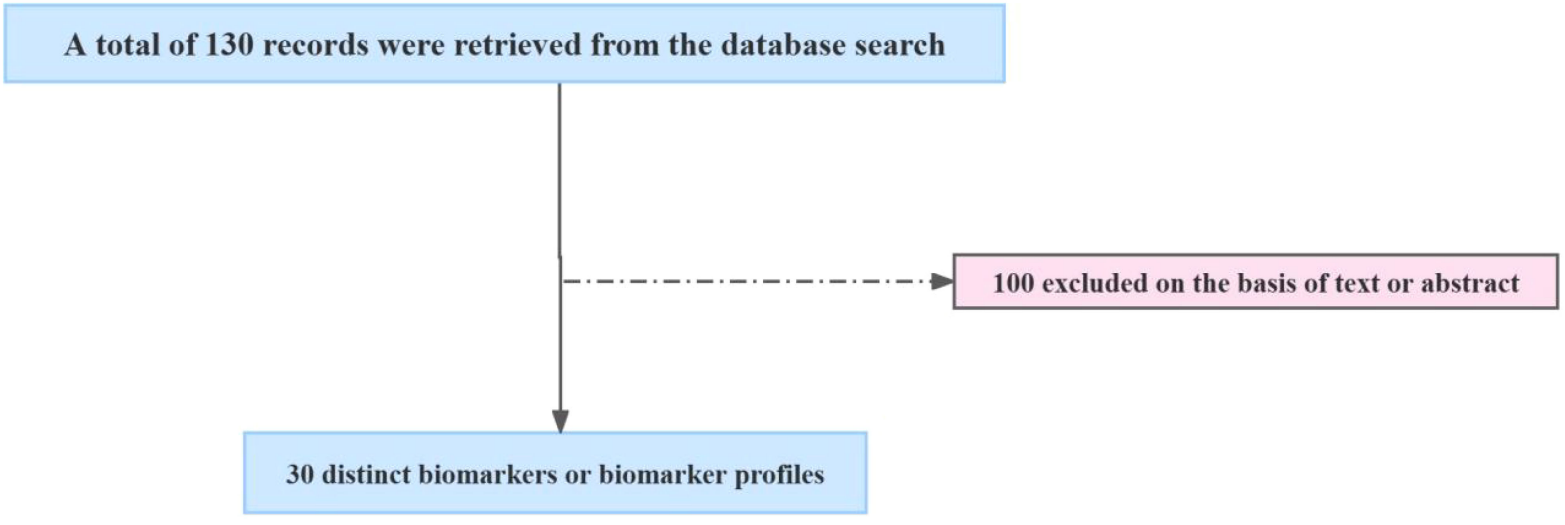
Flowchart depicting the publication screening process.

We conducted a comprehensive literature search using the keywords “diabetes mellitus AND tuberculosis AND biomarkers” in the NCBI - PubMed database, which yielded a total of 130 articles until April 30, 2025. The inclusion criteria for the studies were as follows: (1) the study must focus on biomarkers related to both diabetes mellitus and tuberculosis; (2) the study must provide original experimental data; (3) the study must be published in English. The exclusion criteria included: (1) studies that did not provide sufficient data on biomarkers; (2) studies that focused solely on either diabetes mellitus or tuberculosis without addressing comorbidity; (3) review articles and case reports. After the initial screening, 30 relevant experimental studies were identified (**Figure 1**). These studies were systematically summarized and evaluated for their contribution to the understanding of biomarkers associated with DM-TB comorbidity.

## 3 Results

### 3.1 Microbial and metabolic biomarkers

Recent advancements in metabolomics and microbiomics have identified several microbial and metabolic biomarkers that hold significant promise for the diagnosis and management of tuberculosis in the context of diabetes mellitus.In the realm of microbial biomarkers, specific gut microbiota genera have been identified as potential indicators of latent tuberculosis infection (LTBI) in patients with poorly controlled diabetes. These genera include *Prevotella_9*, *Streptococcus*, *Actinomyces*, *Bacteroides*, *Alistipes*, and *Blautia*. These microbial markers have demonstrated a high degree of accuracy in predicting LTBI status, achieving an area under the receiver operating characteristic (ROC) curve of 0.872, thereby underscoring their potential diagnostic utility (15). Wang et al. (2025) (16) revealed significant disruptions in the gut microbiota composition and metabolomic profiles of pulmonary tuberculosis (PTB) and PTB–DM patients. Key findings included reduced α- and β-diversity of gut microbiota, with specific genera such as g-*Roseburia*, g-*Ruminococcaceae_UCG.013*, and g-*Lachnospiraceae_unclassified* being significantly diminished in PTB–DM patients.

Additionally, untargeted metabolomics identified significant alterations in amino acid metabolism, including elevated levels of serine, proline, and histidine. Metabolomic studies have revealed that lipid, amino acid, and carbohydrate metabolisms are dysregulated in both TB and DM, with potential biomarkers such as kynurenine pathway metabolites and short-chain fatty acids (SCFAs) showing promise for early diagnosis and treatment (17). Studies measuring plasma eicosanoid levels, including LXA4, 15-epi-LXA4, LTB4, and PGE2, have shown that these mediators are significantly altered in patients with TB-DM. Elevated levels of LXA4 and 15-epi-LXA4 correlate with disease severity and bacterial burden, indicating their potential as biomarkers for disease progression and treatment response (18). Studies have also identified a pro-atherogenic lipid profile characterized by increased sphingomyelins and remnant-like lipoprotein particles in TB-DM patients (19). Metabolomic and transcriptomic analyses have underscored the importance of IL-17, PI3K-AKT signaling pathway, and PPAR signaling pathway in the pathogenesis of TB-DM comorbidity (20). This finding suggests that lipid profiling could serve as a valuable tool for assessing cardiovascular risk in patients with comorbid TB and DM (21), emphasizing the need for further research to validate the specific metabolite biomarkers in larger cohorts (22, 23). Therefore, specific gut microbiota genera such as *Prevotella_9*, *Streptococcus*, and *Bacteroides*, as well as lipid mediators like kynurenine pathway metabolites, have been identified as potential biomarkers for both diabetes mellitus and tuberculosis comorbidity, showing promise for early diagnosis and treatment.

### 3.2 Immunological biomarkers

Studies have illuminated significant changes in the immune profiles of patients with tuberculosis and diabetes mellitus comorbidity, particularly in the frequencies and functions of key immune cells. Investigations into the immunological landscape of DM-TB co-infection have revealed that CD8^+^ T cells and NK cells exhibit altered cytokine production and cytotoxic potential. Elevated frequencies of CD8^+^ T cells expressing IFN-γ, IL-2, and IL-17F, as well as NK cells expressing TNF-α and IL-17A, have been observed in TB-DM patients. These elevated frequencies suggest that these immune cells could serve as biomarkers for disease severity and treatment monitoring, with high sensitivity and specificity for identifying active disease (24). This finding underscores the potential of these immune cells as indicators of disease progression and treatment response. The influence of type 2 diabetes (T2DM) on CD8^+^ T-cell responses in latent Mycobacterium tuberculosis infection has also been examined. Research indicates that diabetes is associated with reduced frequencies of CD8^+^ T cells expressing cytokines (Th1, Th2, Th17) and increased expression of cytotoxic markers. This advocates that diabetes may impair immune responses, contributing to increased susceptibility to tuberculosis. The findings bring to the fore the potential of CD8^+^ T cell markers as indicators of immune dysfunction in this comorbidity, with implications for early diagnosis and treatment (25).

Some researches also have explored the function of innate lymphoid cells (ILCs) in TB-DM co-infection. Studies have shown that ILC subsets and their cytokine production are altered in patients with both conditions. Notably, elevated IL-22 production in ILC3 has been observed in TB-DM patients, suggesting that ILCs could be targeted to modulate immune responses in co-infected individuals. This hints at the potential of ILCs as biomarkers for disease progression, with the ability to identify immune dysregulation in comorbid TB and DM (26). The expression of Th1/Th17 cytokines, cytotoxic markers, and immune markers in gamma-delta (γδ) T cells has been investigated in latent tuberculosis patients with diabetes and pre-diabetes. Investigations have found that diabetes and pre-diabetes are associated with reduced expression of these markers in γδ T cells, indicating compromised immune function. This underscores the role of γδ T cells in the pathogenesis of LTB and diabetes comorbidity, with potential as biomarkers for immune status (27).

The frequency of natural-killer T cells (NKT cells) has been examined in peripheral blood and bronchoalveolar lavage fluid of pulmonary tuberculosis patients with and without type 2 diabetes mellitus. NKT cells have been found to be significantly increased in TB patients with DM, suggesting their potential as diagnostic markers for active TB (28). Monocyte surface markers, such as CCR2, CD11b, and RAGE, have been associated with altered monocyte function and TB susceptibility in DM2 patients (29). Targeting CCR2 may enhance the immune response and improve treatment outcomes in patients with comorbid TB and DM. In the aggregate, the identification of altered immune cell profiles, including CD8^+^ T cells, NK cells, ILCs, γδ T cells, monocytes, and NKT cells, offers valuable insights into the pathogenesis of DM-TB and signifies potential biomarkers for diagnosis, treatment monitoring, and therapeutic intervention.

In addition,Ye et al. (2025) (30) conducted a comprehensive study that mined transcriptome data from the GEO database and utilized weighted gene co-expression network analysis (WGCNA) combined with ten machine learning algorithms to identify immune biomarkers associated with DM–TB. The study identified three key immune-related biomarkers—CETP (cholesteryl ester transfer protein), TYROBP (TYRO protein tyrosine kinase binding protein), and SECTM1 (secreted and transmembrane protein 1)—which were used to construct an early alert model for DM–TB. This model demonstrated significant predictive efficiency with an AUC of 0.86 in the training set and 0.901 in the validation set. The identified biomarkers and the constructed model provide a new strategy for early screening and risk prediction of DM–TB, highlighting the potential of immune-related markers in managing this complex comorbidity. Hence,elevated frequencies of CD8+ T cells and NK cells, which express cytokines such as IFN-γ and IL-17, have been observed in patients with DM-TB comorbidity, indicating their potential as biomarkers for disease severity and treatment monitoring.

### 3.3 Inflammatory response biomarkers

The interplay between tuberculosis and diabetes mellitus has been shown to significantly alter the inflammatory response in affected individuals. Elevated levels of inflammatory markers such as C-reactive protein, ferritin, and hepcidin have been consistently observed in patients with DM-TB comorbidity. These markers not only reflect the heightened inflammatory state but also hold potential for monitoring disease severity and guiding treatment strategies. Pre-diabetes has been associated with elevated systemic levels of pro-inflammatory cytokines, which may exacerbate TB pathology (31). Elevated ferritin and hepcidin levels have been identified as indicators of altered iron metabolism in TB patients with diabetes. These changes are associated with increased disease severity and could serve as valuable biomarkers for monitoring the progression of DM-TB comorbidity (32). The modulation of iron status is critical, as it influences both the host’s immune response and the pathogen’s ability to thrive. Intermediate hyperglycemia and diabetes have been shown to significantly alter the host transcriptome in TB patients, characterized by heightened inflammatory responses and reduced type I interferon responses. These alterations suggest that metabolic dysregulation exacerbates immune dysfunction, potentially worsening TB outcomes (33). This alludes to the importance of addressing glycemic control as part of the management strategy for DM-TB comorbidity.

Furthermore, higher CD4 and CD8 cell counts have been observed in TB patients with diabetes compared to those with non-tuberculous pneumonia and healthy controls (34). This points to that diabetes alters the immune response to TB, potentially increasing susceptibility and complicating treatment. Serum levels of chemokines IP-10, IL-8, and SDF-1 have been identified as potential biomarkers for the diabetes-TB nexus (35). Altered levels of these chemokines can impact anti-TB immunity, indicating their potential for monitoring disease progression and treatment response in TB-DM comorbidity. Reduced capacity to inhibit the growth of Mycobacterium tuberculosis has been observed in patients with Type 2 Diabetes Mellitus, particularly those with poor glycemic control (36). This reduced capacity is linked to altered cytokine production, which may contribute to increased susceptibility to TB. The Mycobacterial Growth Inhibition Assay (MGIA) has emerged as a potential *in vitro* marker for assessing immunological control of *M. tuberculosis* in DM2 patients, offering insights into the development of targeted therapies.

In patients with active pulmonary tuberculosis (APTB), elevated levels of the monocyte to high-density lipoprotein cholesterol ratio (MHR), neutrophils to high-density lipoprotein cholesterol ratio (NHR), C-reactive protein-to-lymphocyte ratio (CLR), and C-reactive protein-to-albumin ratio (CAR) have been identified as potential indicators of type 2 diabetes mellitus risk (37). These biomarkers could facilitate early detection and management of DM2 in APTB patients, enhancing overall treatment efficacy. Moreover, Zhang et al. (2025) (38) assessed a range of inflammatory markers in 276 diabetic patients and 276 patients with diabetes mellitus combined with active tuberculosis (DM-PTB) from Kunming, China. The study identified several inflammatory markers, including the neutrophil-to-lymphocyte ratio (NLR), platelet-to-neutrophil ratio (PNR), platelet-to-monocyte ratio (PMR), monocyte to high-density lipoprotein ratio (MHR), and monocyte-to-lymphocyte ratio (MLR), as significant predictors of PTB in diabetic patients. A predictive model combining these markers demonstrated high sensitivity (75.0%) and specificity (81.9%) for identifying diabetic patients susceptible to Mycobacterium tuberculosis infection. Thus, elevated C-reactive protein and ferritin levels are consistently observed in patients with DM-TB comorbidity, reflecting heightened inflammation and serving as potential biomarkers for monitoring disease progression.

### 3.4 Clinical and diagnostic biomarkers

Optimal drug exposure thresholds have been identified as crucial for improving treatment outcomes in multidrug-resistant TB patients with diabetes. In the study of multidrug-resistant tuberculosis (MDR-TB) and diabetes mellitus comorbidity, significant advancements have been made in understanding the pharmacokinetic (PK) and pharmacodynamic (PD) parameters that influence treatment outcomes (39). Specifically, the AUC/MIC (Area Under the Curve/Minimum Inhibitory Concentration) ratios of various anti-TB drugs have emerged as crucial biomarkers for predicting treatment success and guiding therapeutic strategies. Poor glycemic control (defined as HbA1c ≥7%) was found to significantly reduce the drug exposure (AUC) of several key anti-TB medications, including moxifloxacin, linezolid, bedaquiline, and cycloserine. This reduction in drug exposure can lead to suboptimal treatment efficacy and increased risk of treatment failure. The study identified specific AUC/MIC thresholds that are predictive of favorable treatment outcomes: an AUC/MIC ratio of ≥67 for moxifloxacin was associated with a high sensitivity (97.3%) and specificity (90.0%) in predicting successful treatment outcomes, while an AUC/MIC ratio of ≥245 for bedaquiline was predictive of 6-month sputum culture conversion with a sensitivity of 96.8% and specificity of 100%. These ratios serve as important biomarkers for optimizing treatment regimens in patients with MDR-TB and DM. By monitoring these ratios, clinicians can adjust drug dosages to ensure adequate drug exposure, thereby improving treatment outcomes and reducing the risk of drug resistance. The findings suggest that therapeutic drug monitoring (TDM) should be considered for patients with MDR-TB and DM to ensure that AUC/MIC ratios reach the identified thresholds, helping to individualize treatment plans and improve the management of this complex comorbidity.

Elevated serum tumor markers, such as CA-125, CA19-9, and CEA, have been proposed as diagnostic indicators for pulmonary tuberculosis (PTB), particularly in patients with diabetes. CA-125 showed high diagnostic performance in PTB patients with diabetes. Specifically, for PTB patients with type 2 diabetes (TB-DM-IT group), CA-125 had a sensitivity of 88.14% (95%CI: 75.0%–91.6%) and a specificity of 95.83% (95%CI: 85.7%–99.9%), with an AUC of 0.963 (95%CI: 0.907–0.990) (40). CA19–9 and CEA showed relatively lower diagnostic performance compared to CA-125, with CA19–9 having a sensitivity of 77.59% and specificity of 60.47% in TB-DM-IT patients, and CEA having a sensitivity of 83.05% and specificity of 60.98% in the same group. In a study involving 246 cases (113 TB, 59 DM, 74 TB+DM) and 133 controls, MPV and PCT levels were significantly altered in TB+DM patients compared to those with TB or DM alone. MPV showed a specificity of 66.1% (DM vs TB+DM) and a sensitivity of 64.9% (DM vs TB+DM), while PCT demonstrated a specificity of 59.4% (DM vs TB+DM) and a sensitivity of 69.5% (DM vs TB+DM) (41). The study indicated that MPV has a specificity of 66.1% and a sensitivity of 64.9% in distinguishing DM from TB-DM when combined with other clinical parameters.

**Figure 2:**
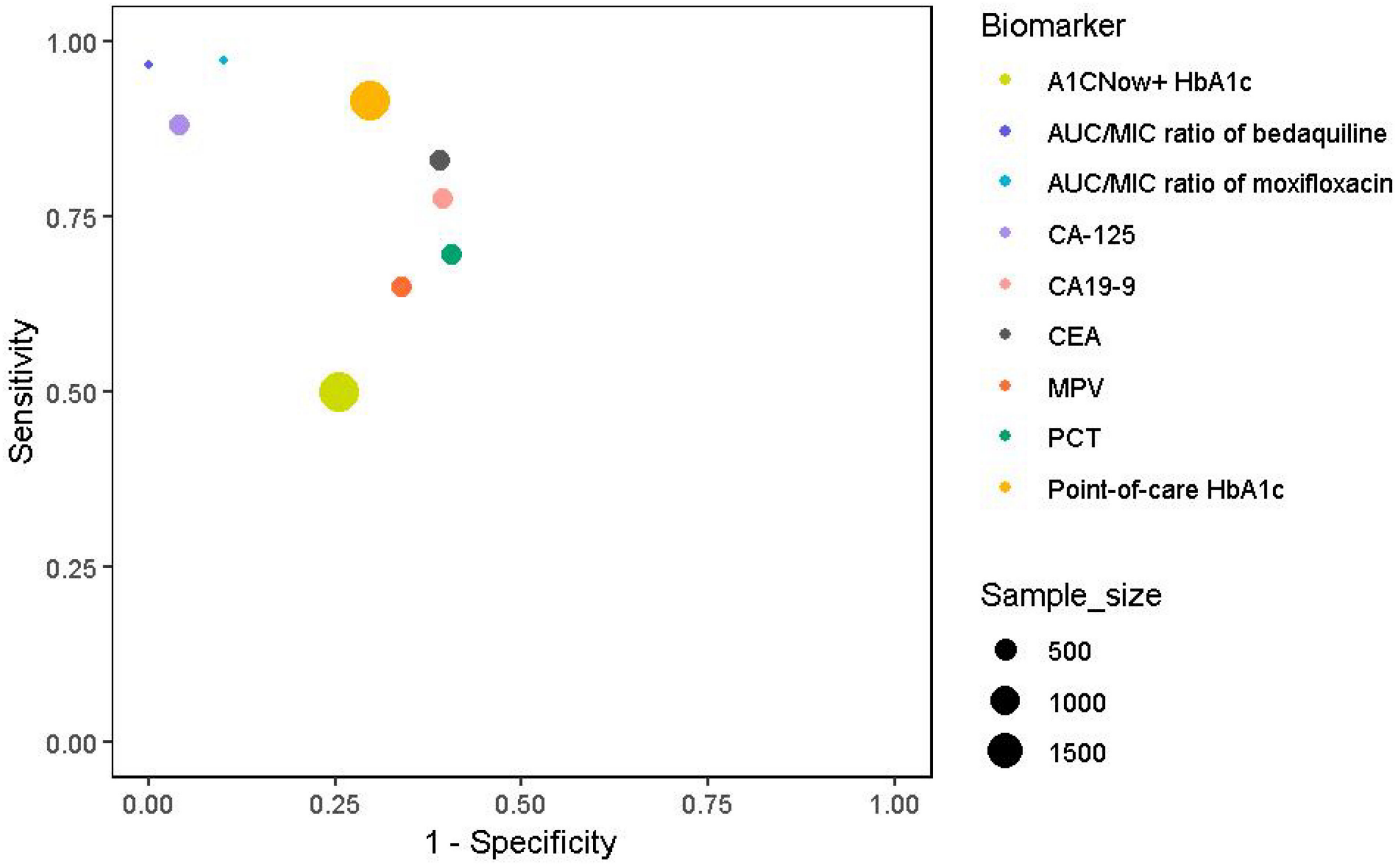
Clinical and diagnostic biomarkers with sensitivity, specificity, and sample sizes. Symbol colours represent different biomarker categories, and the size of the markers represents the study sample size: MPV, PCT (379), Point-of-care HbA1c, A1CNow+ HbA1c (1939), AUC/MIC ratio of moxifloxacin and AUC/MIC ratio of bedaquiline (131), CA-125, CA19-9, and CEA(416).

Furthermore, a two-step screening method, involving random plasma glucose testing followed by point-of-care HbA1c, demonstrated a specificity of 70.4% and a sensitivity of 91.7% for identifying DM in newly diagnosed pulmonary TB patients, imply its effectiveness in resource-limited settings (42). It reported a specificity of 74.5% and a sensitivity of 50.0% for detecting DM using the A1CNow+ system with an HbA1c cutoff of ≥6.5% among TB patients, indicating its potential utility in screening for DM in this population. In tandem, the integration of these biomarkers into clinical practice, as shown in **Figure 2**; **Table 1**, could significantly enhance the diagnosis and treatment of TB, particularly in patients with comorbid diabetes. Accordingly, serum CA-125, with a sensitivity of 88.14% and specificity of 95.83%, and AUC/MIC ratios of anti-TB drugs like moxifloxacin (≥67) have been identified as highly accurate biomarkers for diagnosing and managing DM-TB comorbidity.

**Table 1:** Summary of clinical and diagnostic biomarkers along with their respective sensitivity, specificity, and the number of samples analyzed.

Biomarker(s)	Sensitivity (%)	Specificity (%)	Sample size
MPV	64.9	66.1	379
Point-of-care HbA1c	91.7	70.4	1939
PCT	69.5	59.4	379
A1CNow+ HbA1c	50	74.5	1939
AUC/MIC ratio of moxifloxacin	97.3	90	131
AUC/MIC ratio of bedaquiline	96.8	100	131
CA-125	88.14	95.83	416
CA19-9	77.59	60.47	416
CEA	83.05	60.98	416

### 3.5 Genetic and molecular biomarkers

Progress in bioinformatics and proteomics have illuminated the genetic and molecular underpinnings of the comorbidity between DM and TB, offering crucial insights into the shared pathophysiological mechanisms of these diseases. Specifically, bioinformatics analyses have identified key hub genes associated with both DM and TB, which hold promise as potential biomarkers for disease status and therapeutic targets. These hub genes, including STAT1, IFIT3, RSAD2, IFI44L, and XAF1, were identified through comprehensive network analysis and validated using reverse transcription-quantitative polymerase chain reaction (RT-qPCR) (43). Moreover, further investigation into the regulatory mechanisms involving these hub genes revealed the potential role of specific miRNAs. Seven miRNAs—miR-3680-3p, miR-3059-5p, miR-629-3p, miR-29b-2-5p, miR-514b-5p, miR-4755-5p, and miR-4691-3p were identified as having significant interactions with the hub genes. These miRNAs may play a crucial role in regulating the expression of the hub genes, thereby influencing the pathogenesis of DM and TB comorbidity.

In a complementary approach, proteomics has been employed to analyze plasma proteins in adult patients with active pulmonary tuberculosis and diabetes mellitus. This research has identified several differentially expressed proteins, including haptoglobin and clusterin, which may serve as potential biomarkers linking PTB and diabetes.These findings showcase the importance of understanding the molecular basis of PTB and diabetes comorbidity, paving the way for targeted interventions and improved treatment outcomes (44). Consequently, hub genes such as STAT1 and IFIT3, along with specific miRNAs like miR-3680-3p and miR-3059-5p, have been identified as potential genetic biomarkers associated with both DM and TB, highlighting their role in the pathogenesis of the comorbidity.

## 4 Discussion

The identification and validation of biomarkers for diabetes mellitus and tuberculosis comorbidity represent a critical step forward in addressing the complex interplay between these two diseases (45). As highlighted in our review, the integration of diverse biomarker categories offers a comprehensive approach to improving diagnostic accuracy and therapeutic strategies. However, the translation of these biomarkers into clinical practice faces several challenges that must be addressed to ensure their effective implementation.

One of the primary challenges is the heterogeneity of patient populations affected by DM-TB comorbidity. This includes individuals living with HIV, those with extrapulmonary TB, and pediatric patients, each presenting unique diagnostic and therapeutic challenges (46). For instance, the performance of many biomarkers in smear-negative TB, extrapulmonary TB, and HIV co-infected individuals often falls short of the World Health Organization target product profiles (TPPs) for diagnostic accuracy (47). This underscores the need for biomarkers that can be validated across diverse populations and settings, ensuring their applicability in real-world scenarios. The use of international consortia, such as the Regional Prospective Observational Research for Tuberculosis (RePORT) International consortium, can facilitate standardized sample collection and laboratory protocols, thereby reducing variability and enhancing the reliability of biomarker validation.

Moreover, the validation and implementation of biomarkers require rigorous study designs and standardized protocols. Many studies identified in our review lack the necessary sample sizes and controls to ensure robust validation. The use of international consortia can address this issue by enabling the pooling of resources and expertise, facilitating the discovery and validation of biomarkers (48). Additionally, leveraging multi-omics techniques, such as proteomics, metabolomics, and transcriptomics (17), can help identify comprehensive biomarker signatures that capture the complexity of DM-TB comorbidity. These approaches can provide a more nuanced understanding of the disease mechanisms and improve the accuracy of diagnostic tools.

Another critical aspect is the development of low-cost, point-of-care diagnostic tools that can be easily implemented in resource-limited settings, as emphasized by the WHO TPPs. The integration of advanced technologies, including next-generation sequencing and metabolomics, holds promise for discovering novel markers. Standardized protocols for sample collection and analysis are crucial for ensuring reproducibility and facilitating the translation of these biomarkers into clinical practice.

In summary, the integration of biomarkers into clinical practice has the potential to significantly enhance the diagnosis and treatment of TB, particularly in patients with comorbid diabetes. By addressing the challenges associated with biomarker validation and implementation, and by leveraging collaborative efforts and advanced technologies, we can develop more effective diagnostic tools and improve patient outcomes. Future research should focus on conducting validation studies in diverse populations, implementing standardized protocols, and developing point-of-care diagnostics that meet the WHO TPPs. Through these efforts, we can better manage DM-TB comorbidity and contribute to global disease control initiatives.

## 5 Conclusion

**Figure 3:**
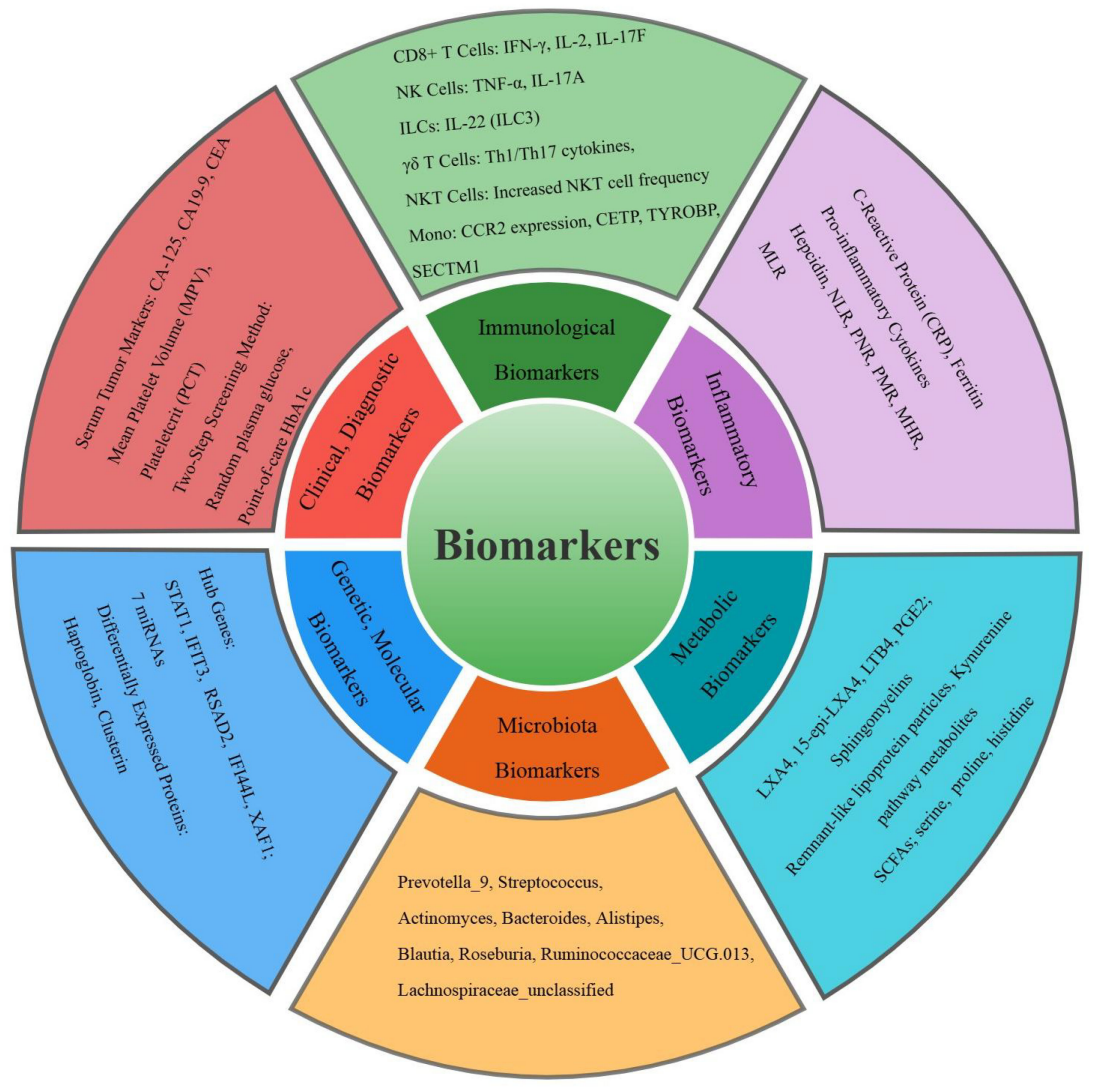
Classification of the common biomarkers for tuberculosis and diabetes mellitus comorbidity in the study. Different colors represent six different categories of biomarkers.

This review synthesizes the current advancements in biomarkers for DM and TB comorbidity, highlighting a spectrum of promising indicators, as indicated in **Figure 3**. Key findings include metabolic markers such as altered gut microbiota genera and lipid mediators, which offer potential for early diagnosis and treatment. Immunological biomarkers like CD8+ T cells and NK cells provide insights into disease severity and treatment monitoring. Inflammatory markers such as elevated CRP and ferritin reflect heightened inflammation and could guide therapeutic strategies. Clinical and diagnostic biomarkers, including serum CA-125 with high sensitivity and specificity, and AUC/MIC ratios of anti-TB drugs like moxifloxacin, demonstrate high diagnostic accuracy. Genetic and molecular biomarkers, including hub genes and miRNAs, present potential therapeutic targets. These biomarkers span microbial, immunological, inflammatory, clinical, and genetic domains, offering a multifaceted approach to improving the diagnosis, monitoring, and treatment of DM-TB comorbidity. Future research should prioritize validating these markers across diverse populations and integrating them into clinical practice to enhance DM-TB management and contribute to global disease control efforts.
